# Supplementary material for: Physiological consequences of Aldolase C deficiency during lactation
Source: PLoS One. 2024 Dec 12;19(12):e0315719. doi: 10.1371/journal.pone.0315719 (PMC11637398; doi:10.1371/journal.pone.0315719)
Supplement: Attachment — Submitted filename: Response to Reviewers.docx [file pone.0315719.s002.docx]

**Response to Reviewers**

**Reviewer #1:** Overall, the study appears to have been well conducted and provided interesting results, but there are issues to consider.

Introduction:
- The purpose of the introduction is to give a scientific background to the main topic of the study.

In the manuscript, the introduction provides detailed background on the role of de novo lipogenesis within the mammary gland as well as the transcriptional regulation of this process and known information on aldolase C.

- The last paragraph of the introduction should state the rationale and hypothesis of the study.

In the revised manuscript, we now provide a concise rationale and hypothesis in the last paragraph of the introduction.

- Line 113: "Deletion of Aldoc did not impact pregnancy or litter size,……" The introduction is not the appropriate place to present the results of the study!!

In the revised manuscript, we have removed specific results and now include an overall statement of findings.

Materials and Methods:
- It is very confusing and makes the results and figures difficult to follow and understand.

In the revised manuscript, we have added relevant information to materials and methods to clarify experimental groups and milk collection time points. The birth dates and milking dates are provided in the supplementary minimal dataset.

- It is advisable to provide a representative flowchart of the study protocol.

A flowchart is included in figure 1D. Additionally, we have added more details to the various groups of mice used in this manuscript.

- Before rushing into the details of biochemical analysis or staining method, you should first clearly define the experimental groups, the number of mice/group and the parameters being measured and in which sample.

In the revised manuscript, we have defined experimental groups, numbers/group, and parameters measured to help clarify.

- Please specify the dates of milk collection and the biochemical parameters being analyzed.

In the revised manuscript, we have included a statement indicating all milk collection was performed within a four-month timeframe and provided exact dates in the supplementary minimal dataset.

- Explain the rationale behind choosing L3, L10 or L14, and why not all parameters are analyzed on the same date.

In the revised manuscript, we have added details and rationale behind choosing lactation day 3 and 10 for milk collection and analysis. As we collected milk from the same mice a multiple time points (L3 and L10), it is not possible to do this on the same date. However, all biochemical milk composition analyses were conducted on same date.

Statistical analysis:
- Did you check for normality of data?

Yes, the data within this manuscript is normally distributed.

- It is not clear which test was used to compare the means of two different groups, and which test was used to compare the means of the same group at different time points (e.g. L3, L10).

We apologize if this wasn’t clear. In the revised manuscript, each figure legend now indicates the statistical test used and is included in the statistical analysis section of the materials and methods section.

- The two-tailed Student's t-test is used to compare two different groups only. Please check the statistical analysis of your data.

For all of our statistical analyses, we are comparing two groups (Aldoc^+/+^ vs Aldoc^-/-^), unpaired two tailed students t-test is the appropriate test for these analyses.

Results:
- Line 197: The word "Aldoa and Aldob" was repeated twice in the same sentence, Please correct.

This is now corrected.

**Reviewer #2:** The manuscript presents valuable and novel findings on the physiological role of Aldoc in lactation. It is well-executed, and the results are robust. To improve the clarity of the manuscript, I would recommend authors to address the following comments:

Abstract:
Consider adding a brief sentence on how these findings could impact broader fields, such as human lactation research.

We now include a sentence on the impact on human lactation within the abstract.

Introduction:
The background on Aldoc's regulatory role in cholesterol and lipid metabolism is relevant. It will benefit from adding a paragraph emphasizing the knowledge gaps about its role in lactation specifically.

In the revised manuscript, we have modified the last paragraph of the introduction to clarify the knowledge gaps, rationale for study, and hypothesis we are testing.

Methods:
The methods section is detailed and well-organized, providing sufficient information for replication. The use of multiple techniques (e.g., lipidomics, histology, and gene expression assays) is a strength. However, the section could benefit from a clearer explanation of how some decisions were made regarding time points for analysis (e.g., why L3 and L10 were chosen).

In the revised manuscript, we now include rational for milk collection at lactation day 3 and 10.

Discussion:
Clarify the distinction between previously published data on Aldoc in other tissues and this study’s findings in the mammary gland and avoid repeating points about metabolic coordination in lactation.

In the revised manuscript, repeated points about metabolic coordination have been removed and previous findings of the role of *Aldoc* in regulating plasma cholesterol and triglycerides have been clarified.

Add a brief conclusion summarizing the implications of Aldoc deficiency in lactation and suggestions for future research directions.

In the revised manuscript, we have added a conclusions section.

Overall, this manuscript presents significant and original research on the role of Aldoc in lactation. After addressing the minor suggestions above, I recommend that this study be published. The work is a valuable contribution to the field of metabolic regulation during lactation.
